# Galectin-1 Ameliorates Influenza A H1N1pdm09 Virus-Induced Acute Lung Injury

**DOI:** 10.3389/fmicb.2020.01293

**Published:** 2020-06-12

**Authors:** Jiaqi Bao, Xiaochen Wang, Sijia Liu, Qianda Zou, Shufa Zheng, Fei Yu, Yu Chen

**Affiliations:** ^1^Department of Laboratory Medicine, The First Affiliated Hospital, College of Medicine, Zhejiang University, Hangzhou, China; ^2^Key Laboratory of Clinical in vitro Diagnostic Techniques of Zhejiang Province, Hangzhou, China; ^3^Institute of Laboratory Medicine, Zhejiang University, Hangzhou, China; ^4^School of Laboratory Medicine and Life Sciences, Wenzhou Medical University, Wenzhou, China; ^5^State Key Laboratory for Diagnosis and Treatment of Infectious Diseases, Collaborative Innovation Center for Diagnosis and Treatment of Infectious Diseases, The First Affiliated Hospital, School of Medicine, Zhejiang University, Hangzhou, China

**Keywords:** influenza virus, H1N1pdm09, galectin-1, acute lung injury, inflammation, replication, apoptosis

## Abstract

Influenza remains one of the major epidemic diseases worldwide. Acute lung injury mainly caused by excessive pro-inflammatory host immune responses leads to high mortality rates in severe influenza patients. Galectin-1, an animal lectin ubiquitously expressed in mammalian tissues, is reported to play important roles in viral diseases. Here, we established murine and A549 cell models to explore the potential roles of galectin-1 treatment in H1N1pdm09-induced acute lung injury. We found that galectin-1 protein level was elevated in A549 cell culture supernatants and mouse BALF after H1N1pdm09 challenge. *In vivo* experiments showed recombinant galectin-1 treatment reduced wet/dry weight ratio, inflammatory cell infiltration in mouse lungs and mediated the expression of cytokines and chemokines including IL-1β, IL-6, IL-10, IL-12(p40), IL-12(p70), G-CSF, MCP-1, MIP-1α and RANTES in serum and BALF of infected mice. Reduced apoptosis and viral titers in mouse lungs were also found after galectin-1 treatment. As expected, galectin-1 treated mice performed reduced body weight loss and enhanced survival rate against H1N1pdm09 challenge. In addition, *in vitro* experiments showed that viral titers decreased in a dose-dependent manner and cell apoptosis in A549 cells reduced after recombinant galectin-1 treatment. Taken together, our findings indicate a potentially positive effect of Gal-1 treatment on ameliorating the progress of H1N1pdm09-induced acute lung injury and recombinant galectin-1 might serve as a new agent in treating influenza.

## Introduction

Influenza is a major public health concern, yearly causing about 250,000–500,000 deaths worldwide, and has not been restrained so far (Thompson et al., 2018). Among three influenza virus types to which humans are susceptible, influenza A virus (IAV) is reported in most areas and is one of the most common causes of severe acute pneumonia and pneumonia-related death (Herold et al., 2015). In early 2009, a triple reassortant strain of the human influenza A (H1N1) subtype (H1N1pdm09) crossed the species barrier from swine to humans and caused severe outbreaks (Yamayoshi and Kawaoka, 2019). Up to now, H1N1pdm09 has gradually replaced the old lineages and circulated yearly in the human population (Hussain et al., 2017).

IAV-associated deaths are mainly attributed to acute lung injury (ALI) and its severe form, acute respiratory distress syndrome (ARDS) (Hussain et al., 2017), which is often found in severe and dead cases of H1N1pdm09 patients (Herold et al., 2015). Some evidence demonstrates that the incidence of seasonal influenza- associated ALI and ARDS at has been estimated at 2.7 cases per 100,000 persons per year and is responsible for about 4% of all hospitalizations for respiratory failure during the influenza season (Peteranderl et al., 2016). The mechanism by which IAV infection induces ALI in the host is complicated. Current studies indicate that ALI may result from the combination of efficient viral replication and robust host immune response in the lower respiratory tract (Liu et al., 2019; Zhao et al., 2020). During IAV infection, viral replication in target cells causes cell damage, which may induce lung injury. Whereas, host pro-inflammatory immune response induced by viral invasion can act as a “double-edged sword” (Newton et al., 2016). On the one hand, inflammatory mediators recruit immune cells to the infected site for viral clearance; on the other hand, excessive activation of immune response aggravates lung tissue injury, which can accelerate the progress and worsen outcomes of the disease, and be considered as the primary cause of ALI (Newton et al., 2016). Thus, such therapeutic strategies aiming to reduce an unbalanced, overshooting inflammation, which have good effects on IAV-induced ALI, may be an alternative approach for the treatment of IAV infection (Herold et al., 2015).

Galectin-1 (Gal-1), a member of an evolutionarily conserved glycan-binding protein family (Sundblad et al., 2017), is reported to be ubiquitously distributed in mammalian tissues (Vasta, 2009) and can function as a soluble multifunctional protein (Garner et al., 2015). Various essential functions of Gal-1 were found in modulating biological processes including cell proliferation, apoptosis (Shih et al., 2019), immune homeostasis, inflammation (Sundblad et al., 2017) and tumor metastasis (Vasta, 2009). In recent years, accumulated reports have revealed that Gal-1 can be up- or down- regulated by virus infection, prompting the potential role of Gal-1 in regulating virus pathogenesis and viral diseases (Vasta, 2009; Lee et al., 2015). Some researches demonstrated that Gal-1 could extracellularly bind to viral proteins or modulate intracellularly pathways (Nita-Lazar et al., 2015b; Wang et al., 2019), and the regulation processes vary among various viruses. Garner et al. found that Gal-1 enhanced Nipah virus attachment to primary human endothelial cells (Garner et al., 2015). Levroney et al. (2005) proved the inhibition function of Gal-1 in envelope glycoproteins of Hendra virus fusion with targeted cells. Other two researches revealed that Gal-1 can bind to influenza virus and *pneumococcal* when coinfecting with influenza virus (Yang et al., 2011; Nita-Lazar et al., 2015a). Meanwhile, critical functions of Gal-1 in regulating virus infections can also occur by interacting with immune cells and modulating related cytokines (Wang et al., 2019). Current evidence reveals that Gal-1 displays broad anti-inflammatory activity in viral infections or viral induced tissue injuries (Sundblad et al., 2017), which may finally ameliorate viral disease outcomes. For example, Gal-1 inhibits proliferation and interferon-γ (IFN-γ) expression in Epsteine-Barr virus (EBV) infection (Gandhi et al., 2007) and diminishes the severity of herpes simplex virus 1 (HSV-1) induced ocular immunopathological lesions (Rajasagi et al., 2012). However, the functions of Gal-1 in alleviating H1N1pdm09-induced acute lung injury, modulating related cytokines and chemokines and its value in influenza therapy are not completely determined.

Here, we established H1N1pdm09-infected murine and A549 cell models to explore the potential role of Gal-1 in modulating lung damage severity and the level of inflammation in H1N1pdm09-induced ALI.

## Materials and Methods

### Cells and Mice

Madin-Darby canine kidney (MDCK) cells were purchased from the American Type Culture Collection (ATCC) and were routinely cultured in Dulbecco modified Eagle medium (DMEM) (Gibco, CA, United States) supplemented with 10% fetal bovine serum (FBS), penicillin (100 U/mL) and streptomycin (100 μg/mL) (Gibco, CA, United States). The human lung adenocarcinoma (A549) cells were cultured in RPMI 1640 medium (Gibco, CA, United States) supplemented with 10% FBS (Gibco, CA, United States) and penicillin/streptomycin (100 U/mL, 100 μg/mL) (Gibco, CA, United States). Both the MDCK and A549 cells were cultured at 37°C and in a humidified atmosphere of 5% CO_2_.

4-to 5-week-old female C57BL/6 mice were used in this study. Following transportation, mice were acclimatized for 7 days before randomized into experimental cages. For mice feeding, water and the same type of food pellets (irradiated standard mouse chow) were provided *ad libitum*. All experiments were performed in compliance with ethical requirements and were approved by the Research Ethics Committee of the First Affiliated Hospital, Zhejiang University School of Medicine.

### Virus and Virus Culture

A/California/04/2009 and mouse-adapted A/California/04/2009 were used in this research. For virus amplification, MDCK cell monolayers were maintained in DMEM supplemented with 0.2% bovine serum albumin (BSA), 25mM HEPES buffer solution (Gibco, CA, United States), tolylsulfonyl phenylalanyl chloromethyl ketone treated (TPCK) trypsin (2 μg/mL) (Sigma, Germany), penicillin (100 U/mL), and streptomycin (100 μg/mL) (Gibco, CA, United States). Viruses were inoculated with MDCK cells at 35°C with a humidified atmosphere of 5% CO2 and 95% air. When 70–100% of the cell monolayer exhibited the cytopathic effect (CPE), the cells were frozen and thawed three times and viruses were harvested. The cultured virus stocks were aliquoted and stored at –80°C until use.

### Measurement of Viral Titers

MDCK cells were seeded in 96-well plates at a density of 3 × 10^4^ cells per well. Twenty-four hours later, cells were washed with sterile phosphate buffer solution (PBS) three times and were added with 100 μL serially diluted virus stocks per well and incubated at 37°C for 1 h. After incubation, cells were washed with sterile PBS and 100 μL fresh DMEM containing 0.2% bovine serum albumin (BSA), tolylsulfonyl phenylalanyl chloromethyl ketonetreated (TPCK) trypsin (2 μg/mL) (Sigma, Germany), penicillin (100 U/mL) and streptomycin (100 μg/mL) (Gibco, CA, United States) was added. Then cells were cultured for 72 h and 50 μL of cultured supernatants was incubated with equal volume of PBS-washed 1% chicken erythrocytes for 45 min. 50% tissue culture infectious dose (TCID_50_) of the virus supernatants were calculated by the Reed–Muench method.

### Cell and Animal Studies

A549 cells were seeded in 6-well plates, exposed to H1N1pdm09 under serum-free conditions for 1 h and then cultured with fresh PRMI 1640 medium (without FBS) for 12, 24, 36, 48, and 72 h. For Gal-1 treatment experiments, A549 cells were incubated with H1N1pdm09 (1 × 10^3^ TCID_50_) for 2 h and recombinant human Gal-1 (1, 5, and 10 μg/mL) was added into the wells. At 24 or 72 h post-infection (h.p.i.), cells and supernatants were harvested for further examination.

Mice were intranasally inoculated with influenza viruses (1.5 × 10^4^ TCID_50_) with a volume of 100 μL (50 μL/nostril) diluted in sterile PBS at 0 day post-infection (d.p.i.). Two and four days later, the mice were treated with recombinant mouse Gal-1 (50 μg/mouse) or equal volume of sterile PBS via the nasal route (2 and 4 d.p.i.). After 2 days, the mice were euthanized for harvesting of tissues, using sterile techniques (6 d.p.i.).

### Analysis of Mouse Body Weight and Survival Rates

Mice were weighed and monitored, at least daily, for illness and mortality. Mice found to be moribund were euthanized and were considered to have died that day. The weight loss of each mouse and the rates of survival of each group were recorded consecutively for 15 days. The results of survival rates of different groups of mice were performed using Kaplan–Meier survival analysis.

### Collection of Bronchoalveolar Lavage Fluid (BALF) and Lung Homogenate Samples

The collection of BALF were performed as previously reported with minor modifications (Xue et al., 2017). Briefly, each lung was gently flushed three times with 0.7 mL sterile saline (0.9%) using a tracheal cannula, the total recovery rate was more than 90%. For the BALF samples without cells, BALF recovered were pooled and centrifuged (400 × *g* for 10 min at 4°C), then the supernatant was collected. All the BALF supernatant was stored in aliquots at –80°C until use. The cell pellet was washed using red blood cell lysis solution. Then cells were centrifuged at 300 × *g* for 10 min and resuspended in 500 μL PBS. The total cell count was determined using a hemocytometer.

The collection of lung homogenate supernatant was performed by homogenizing the lungs in sterile PBS. Homogenates were centrifuged at 3000 rpm for 10 min at 4°C. The supernatant was collected and stored at –80°C (Chen et al., 2018).

### Quantitative Real-Time PCR Analysis

To detect levels of mRNA expression, total RNA from mouse lungs and A549 cells was extracted using QIAGEN RNeasy Mini Kit (QIAGEN, Hilden, Germany). Extrated RNA was reverse transcribed into cDNA using QuantiTect Rev. Transcription Kit (QIAGEN, Hilden, Germany) according to the manufacturer’s instructions. Quantitative real-time PCR was performed using the QuantiFastTM SYBR Green PCR Kit (QIAGEN, Hilden, Germany) with an ABI 7500 instrument (Applied Biosystems, CA, United States). The primer sequences were available on request and were listed in Table 1. All reactions were carried out in triplicate in the same plate. The relative gene expression levels were determined by normalizing the Ct levels of the mRNA of interest to endogenous GAPDH. The ΔΔCt method was applied to evaluate and compare differential gene expression between samples. The data were analyzed using the ABI 7500 software v2.0.5 (Applied Biosystems, CA, United States).

**TABLE 1:** Sequences of primers in quantitative real-time PCR.

Species	Gene name	Forward (5′-3′)	Reverse (5′-3′)
H1N1	H1N1-HA	CATGCGAACAATTCAACAGAC	ATGCTGCCGTTACACCTTT
	H1N1-NP	CTGCTTGTGTGTATGGGCTTG	GCGGATGCCTTCTGTTGATT
	H1N1-M1	CTACGGCAAAGGCTATGG	TGCTCCTGTTGATATTCTTCC
	H1N1-NS1	TTTGCGTGCGATTGGACC	TGTTCCCGCCACTTCTCATTT
Mouse	GAPDH	GCCTCGTCCCGTAGACAAAA	CCCTTTTGGCTCCACCCTTC
	Galectin-1	TGCCTCCATGTGTTCTTGGTC	TGTGGTCACTTAAGCCCTCT
Homo sapiens	Galectin-1	CGGGAACATCCTCCTGGACTCA	CCTCGCACTCGAAGGCACT
	GAPDH	AGAAGGCTGGGGCTCATTTG	AGGGGCCATCCACAGTCTTC

### Enzyme-Linked Immunosorbent Assay (ELISA)

For the determination of mouse Gal-1 protein, serum and BALF of each mouse were collected and the level of Gal-1 was detected using Mouse Galectin-1/LGALS1 ELISA Kit (ORIGENE, Rockville, United States) according to the manufacturer’s protocols. For the analysis of human Gal-1 protein concentration in the medium of A549 cells, culture supernatants were collected at the indicated times and were detected using Human Galectin-1/LGALS1 ELISA Kit (Sino Biological, Beijing, China) according to the manufacturer’s instructions. Absorbance was measured at 450 nm using the SpectraMax i3x detection system (Molecular Devices, CA, United States).

### Lung Wet/Dry Weight Ratios

To assess the extent of acute pulmonary edema, mice were sacrificed and their middle lobe lungs were immediately weighed to obtain the wet weight. Then the lungs were heated in an oven at 80°C for 72 h, the dry weight of the lungs was recorded, after which the wet-to-dry weight ratios were calculated.

### Histological and Immunohistochemical Examination of the Lungs

Lung tissues isolated from mice were immediately fixed in 10% buffered formalin for 48 h at room temperature. Then the tissues were embedded in paraffin for cutting and staining with hematoxylin and eosin (H&E) according to the standard protocols. Serial sections (4-μm thick) were finally examined microscopically (LEICA, Wetzlar, Germany). The distribution of viruses was examined by immunohistochemical staining as previously reported (Ou et al., 2016) with minor modifications. Sections (4-μm thick) of mice lungs were deparaffinized and then subjected to antigen retrieval by heat treatment. Endogenous peroxidase activity was quenched with 0.3% H_2_O_2_ in methanol. Sections were blocked for 90 min with 3% BSA in PBS and incubated overnight with 1:200 dilution of polyclonal rabbit anti-influenza A virus nucleoprotein (NP) antibody (GeneTex, CA, United States) at 4°C, placed in a wet box containing a little water. The next day, secondary antibody (appropriately respond to primary antibody in species) labeled with HRP was added to cover objective tissue, and the slides were incubated at room temperature for 50 min. For DAB developing, sections were dried slightly and fresh prepared DAB chromogenic reagent was added to marked tissue. The slides were counterstained with hematoxylin and the results were reviewed and photographed using LEICA MD 2000 microscope system (LEICA, Wetzlar, Germany). Numbers of NP-positive cells were calculated from 6 randomly selected fields in each section (original magnification, ×400).

### Apoptosis Analysis

Paraffin-embedded lung tissue sections (4-μm thick) were subjected to TUNEL (terminal deoxynucleotidyl- transferasemediated dUTP-biotin nick end labeling) assay according to the manufacturer’s instructions (Roche, Basel, Switzerland). The other method for measuring cell apoptosis was conducted using Cell Death Detection ELISA^PLUS^ (Roche, Mannheim, Germany) according to the manufacturer’s protocol, which is based on an antigen-capture method for histone and fragmented DNA detection.

### MTT Assay

Cytotoxic effect of Gal-1 on the A549 cells was determined by MTT assays using MTT Cell Proliferation and Cytotoxicity Assay Kit (Solarbio, Beijing, China), according to the manufacturer’s protocols. Briefly, A549 cells were seeded into 96-well plates with 3000–10000 cells per well. After being incubated at 37°C for 18 h, the cells were treated with mock or Gal-1 for the indicated time points (24 and 72 h). Control cells were treated only with medium. Then the culture medium was discarded and replaced with 90 μL fresh medium containing 10 μL MTT solution per well (provided in the kit) for another 4 h. Medium was discarded again and replaced with 110 μL provided formazan solution per well, incubating on the shaker at a low speed for 10 min to fully dissolve the crystals. Finally, the absorbance was determined at a wavelength of 490 nm using the SpectraMax i3x detection system (Molecular Devices, CA, United States). For each time point, the treated cells were compared with control cells and the percentage of cell viability was calculated as (mean OD of treated cells/mean OD of control cells) × 100.

### Cytokine and Chemokine Measurement

Cytokines and chemokines in BALF and serum samples from the mice were measured using multiplex bead assays (Bio-Rad, CA, United States) according to the manufacturer’s instructions. For analysis, the 96-well plate was placed in a Bio-Plex reader and the data collection, analysis and quality control were performed as previously described (Hye et al., 2014).

### Transmission electron Microscope (TEM) Analysis

Small blocks (about 1 mm^3^) of lungs were fixed in 2.5% glutaraldehyde and phosphate buffer 0.1 M (pH = 7.4) overnight and then fixed in 1% osmium tetroxide, dehydrated through graded ethanol solutions, and embedded in Epon-Araldite. Ultrathin sections obtained from selected areas were made using an ultramicrotome (Leica EM UC6) and then stained with uranyl acetate and lead citrate. After staining, the sections were observed using a transmission electron microscope (Philips TECNAI 10).

### Statistical Analysis

Data was expressed as means ± the standard deviations (SD). *P*-values between two groups were calculated using the student *t*-test. For more than two groups, *P*-values were calculated using one-way analysis of variance (ANOVA). Data was analyzed using SPSS 19.0 software. For all analyses, *P* < 0.05 was considered statistically significant and all probabilities were two-tailed.

## Results

### H1N1pdm09 Replicates Effectively in A549 Cells and Mouse Lungs and Induces Lung Injury

**FIGURE 1:**
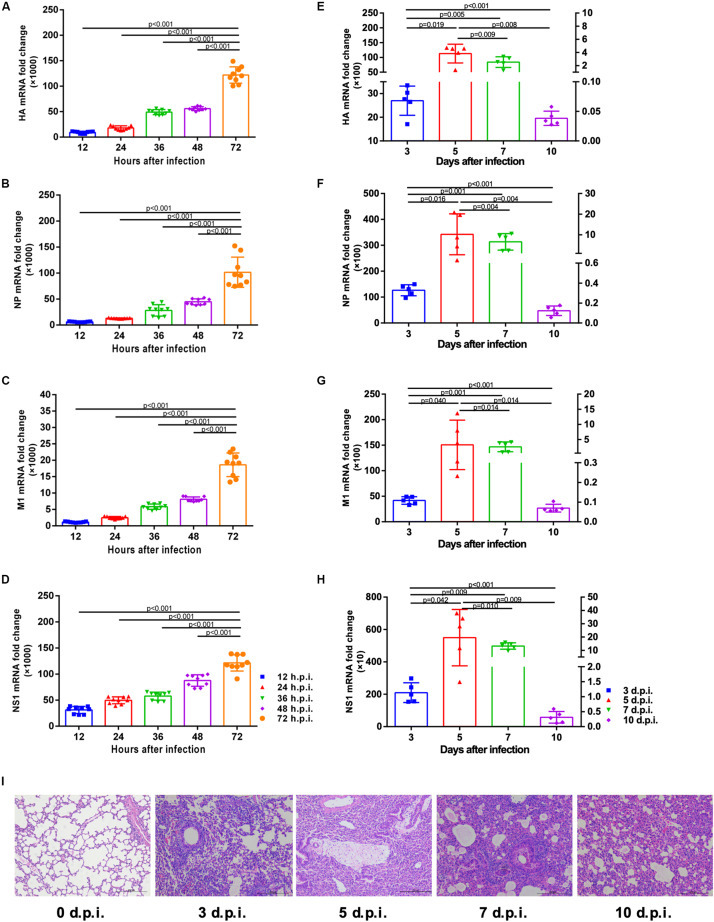
Viral mRNA expression in A549 cells and mouse lungs and pathological changes of mouse lungs at indicated time after H1N1pdm09 infection. **(A–D)**. Viral HA **(A)**, NP **(B)**, M1 **(C)**, and NS1 **(D)** mRNA copies in A549 cells at 12, 24, 36, 48, and 72 h after infection with 1 × 10^3^ TCID_50_ H1N1pam09 (*n* = 9). **(E–H)** Viral HA **(E)**, NP **(F)**, M1 **(G),** and NS1 **(H)** mRNA copies in mouse lungs at 3, 5, 7, and 10 days after infection with 1.5 × 10^4^ TCID_50_H1N1pam09 (*n* = 5; data of 3 and 5 d.p.i.: refer to left *Y* axis; data of 7 and 10 d.p.i.: refer to right *Y* axis). **(I)** H&E-stained lung sections collected at 0, 3, 5, 7, and 10 days after infection (original magnification: ×100, scale bar = 200 μm, *n* = 5). Representative data shown from three independent experiments. *P*-values are indicated in the panels. HA, hemagglutinin; NP, nucleoprotein; M1, matrix protein 1; NS1, non-structural 1 protein.

The correlation between viral mRNA expression and H1N1pdm09 infection was first determined. Results indicated the presence of progressively increased viral HA, NP, M1 and NS1 copies in A549 cells infected by H1N1pam09 at indicated time points (Figures 1A–D). In mouse lungs, mRNA expression of HA, NP, M1, and NS1 revealed a strong increase during the first 5 days after infection and reached a peak around 5 d.p.i. (left *Y* axis). Mice started to clear the infection from around 7 d.p.i. (right *Y* axis) with significantly reduced viral copies (Figures 1E–H; data of 3 and 5 d.p.i.: refer to left *Y* axis; data of 7 and 10 d.p.i.: refer to right *Y* axis). Lung histopathology showed obvious injures in infected mouse lungs with thickened alveolar walls, diminished alveolar lumen and inflammatory cell infiltration, and injuries existed at all indicated time points. In comparison, lungs from uninfected mice had no apparent histopathological lesions (Figure 1I). These results suggested that H1N1pdm09 could replicate effectively in A549 cells and mouse lungs and could lead to typical acute lung injury in mice, which showed a success in the establishment of cell and mouse infection model, laying a foundation for the following experiments.

### Gal-1 Protein Level Is Elevated After Infection With H1N1pdm09

**FIGURE 2:**
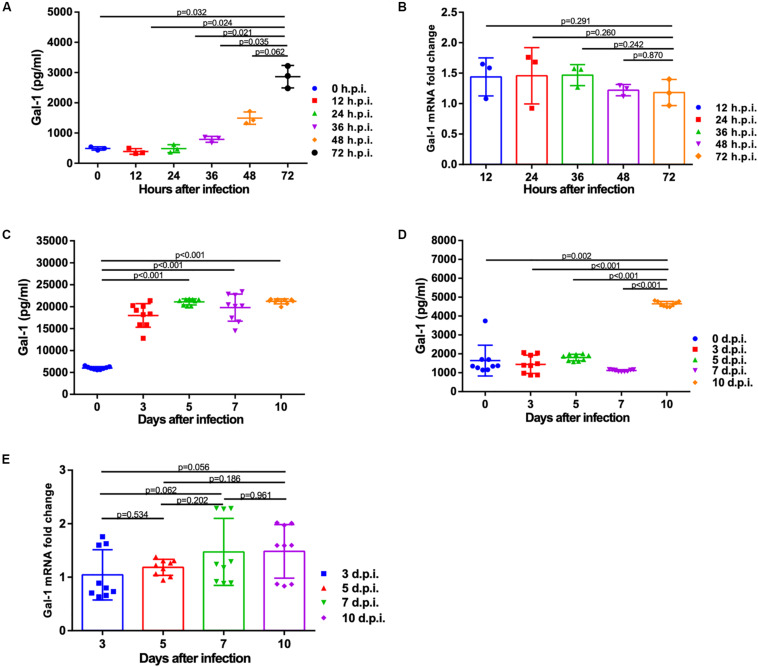
Expression of Gal-1 protein and mRNA after infection with H1N1pdm09 virus. **(A)** Gal-1 protein level in the supernatant of A549 cells at 0, 12, 24, 36, 48, and 72 h after infection with H1N1pdm09. **(B).** The fold change of Gal-1 mRNA level in A549 cells at 12, 24, 36, 48, and 72 h after infection with H1N1pdm09 (*n* = 3). **(C,D)** Gal-1 protein level in mouse BALF **(C)** and serum **(D)** at 0, 3, 5, 7, and 10 days after infection with H1N1pdm09. **(E)** The fold change of Gal-1 mRNA level in mouse lung homogenates at 3, 5, 7, and 10 days after infection with H1N1pdm09 (*n* = 9). Representative data shown from three independent experiments. *P*-values are indicated in the panels. BALF: bronchoalveolar lavage fluid.

To address whether H1N1pdm09 affected the expression of Gal-1, protein and mRNA levels of Gal-1 were quantified. Protein level of Gal-1 was significantly increased in the supernatant of A549 cells at 72 h with the infection of H1N1pdm09 (Figure 2A). Results of animal experiments showed significant up-regulation of Gal-1 protein in mouse BALF from around 3 days infected with N1H1pdm09, while the levels between different time points post infection had no significance (Figure 2C). As shown in Figure 2D, the concentration of Gal-1 in mouse serum reached peak around 10 days after infection and the difference between the time points before 7 d.p.i. was not significant, which was different from the results of BALF. However, the mRNA expression of Gal-1 was not affected by H1N1pdm09 infection at all indicated time points in both A549 cells and mouse lungs (Figures 2B,E). Taken together, Gal-1 protein not mRNA was modulated by H1N1pdm09 infection, suggesting a potential role of Gal-1 protein in the infection of H1N1pdm09.

### Gal-1 Treatment Ameliorates Edema and Pathological Changes in Mice Lungs

**FIGURE 3:**
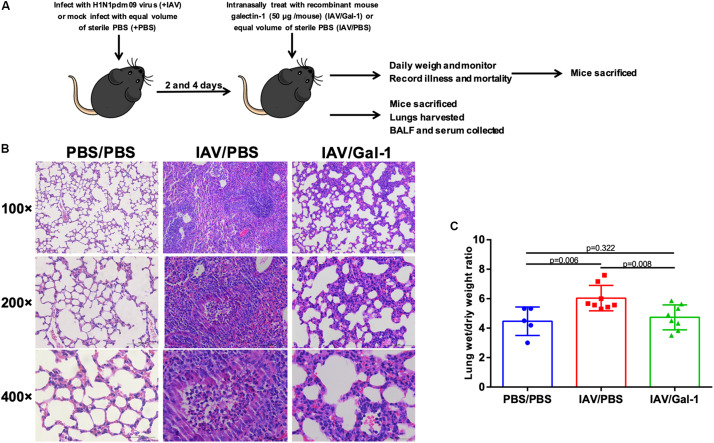
Edema and pathological changes in the lungs of H1N1pdm09-infected mice by Gal-1 treatment. **(A)** 4-to 5-week-old female C57BL/6 mice were infected with H1N1pdm09 (1.5 × 10^4^ TCID_50_) at 0 d.p.i. At 2 and 4 d.p.i., mice were treated with recombinant mouse Gal-1 (50 μg/mouse) or equal volume of sterile PBS via the nasal route. At 6 d.p.i., mice were euthanized for harvesting of samples and examination. For weight loss and survival rate measurement, mice were monitored daily until 15 d.p.i. **(B)** H&E-stained lung sections collected at 6 d.p.i. (original magnification, top panel: ×100, scale bar = 200 μm, middle panel: ×200, scale bar = 100 μm, bottom panel: ×400, scale bar = 50 μm). **(C)** Wet-to-dry ratios of lungs of Gal-1-treated mice compared with untreated controls at 6 d.p.i. (*n* = 5–8). Representative data shown from at least two independent experiments. *P*-values are indicated in the panels.

We next examined the effects of recombinant Gal-1 treatment on the severity of acute injury in mouse lungs after H1N1pdm09 infection. C57BL/6 mice were exposed to H1N1pdm09 virus and were treated with recombinant mouse Gal-1 or PBS at 2 and 4 d.p.i., then the mice were sacrificed and lung tissues were collected (Figure 3A). Histological examination of lungs showed that in IAV/Gal-1 group (Figure 3B, right panel), the destruction of lung tissue was less severe compared with those in IAV/PBS group (Figure 3B, middle panel), with reduced destruction of alveolar structure and inflammatory cell infiltration in the bronchus. Meanwhile, lung wet/dry weight ratio, a direct indicator of pulmonary edema (Davieds et al., 2016), was also examined to evaluate the severity of lung injury. As shown in Figure 3C, after Gal-1 treatment, the lung wet/dry weight ratios reduced significantly compared with the untreated group. Collectively, these results suggested a role of Gal-1 treatment in alleviating lung edema, pathological changes by H1N1pdm09 challenge.

### Gal-1 Treatment Attenuates Lung Apoptosis in H1N1pdm09-Infected Mice

**FIGURE 4:**
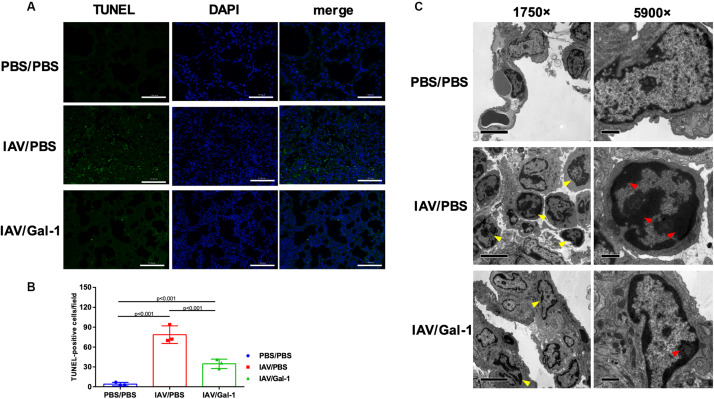
Apoptosis analysis in the lungs of H1N1pdm09-infected mice by Gal-1 treatment. **(A)** Staining of lung sections with TUNEL to detect the apoptotic cells in the lungs collected at 6 d.p.i. (*n* = 3, original magnification, ×200, scale bar = 100 μm; green, TUNEL; blue, DAPI counterstaining of cell nuclei.). **(B)** TUNEL-positive cells in mouse lungs were quantified by averaging the number of positive stained cells in six randomly selected fields in each section (*n* = 3). **(C)** Apoptotic nuclear morphology observed by transmission electron microscopy (*n* = 4, original magnification, left panel: ×1750, scale bar = 5 μm; right panel: ×5900, scale bar = 1 μm; yellow arrow head: chromatin-condensed cells; red arrow head: condensed chromatin). Representative data shown from at least two independent experiments. *P*-values are indicated in the panels. DAPI, 2-(4-amidinophenyl)-6-indolecarbamidine dihydrochloride.

Apoptosis in the lung is a well-described feature of IAV-induced ALI (Herold et al., 2015; Yang et al., 2017). Therefore, we further determined whether Gal-1 treatment also influences H1N1pdm09-induced apoptosis of mouse lungs using TUNEL and TEM assay. As shown in Figure 4A, TUNEL-positive cells (indicated by green fluorescence) were abundantly seen in the IAV/PBS group (Figure 4A, middle panel), whereas less seen in the IAV/Gal-1 group (Figure 4A, bottom panel). Additionally, the number of apoptotic cells in treated group was about half of those in the untreated group, and the difference was significant (Figure 4B). To further confirm the effect of Gal-1 treatment on the extent of cell apoptosis, we examined TEM images of lung samples from different groups. We found that the number of chromatin-condensed cells (indicated by yellow arrow heads) was larger in each field in IAV/PBS group, compared with IAV/Gal-1 group (Figure 4C, left panel, 1750×). Besides, chromatin condensation of cells was more severe in untreated group than Gal-1-treated group. As shown in the right panel of Figure 4C (5900×), markedly condensed chromatin (indicated by red arrow heads) was close to the nuclear membrane in IAV/PBS group, compatible with the apoptotic morphology, while relatively loose chromatin was observed in IAV/Gal-1 group. Taken together, these results demonstrated that Gal-1 treatment could attenuate lung apoptosis in mice, which indicated ameliorated ALI caused by H1N1pdm09.

### Gal-1 Treatment Modulates the Expression of Cytokines and Chemokines and Reduces Inflammatory Cell Infiltration in Infected Mice

**FIGURE 5:**
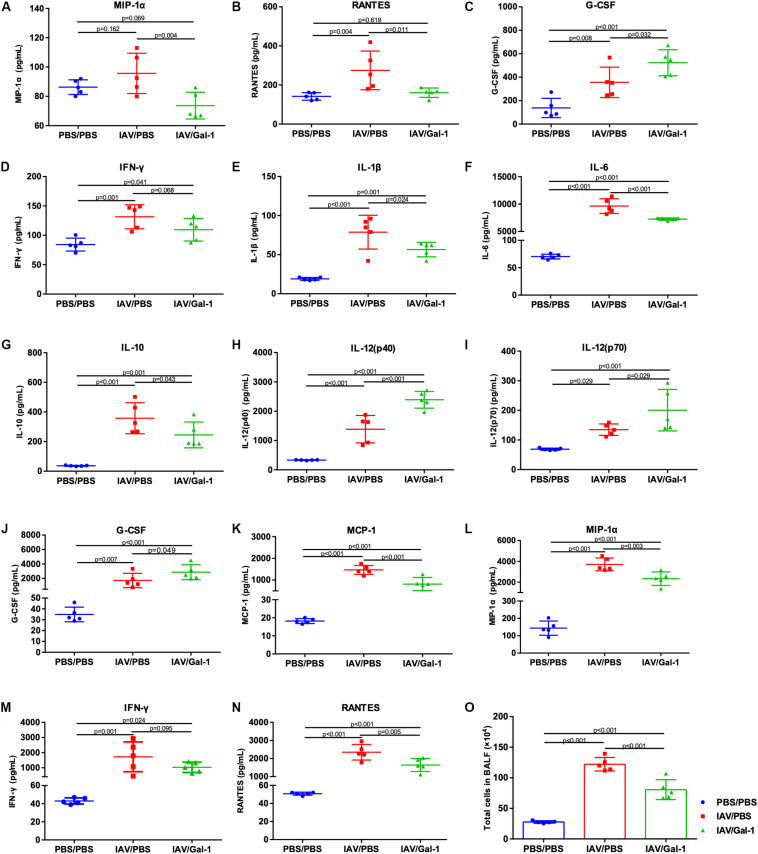
Assessment of cytokines and chemokines and total cell numbers in serum or BALF of H1N1pdm09-infected mice in different groups. **(A–D)** Expression of cytokines and chemokines in mouse serum. **(E–N)** Expression of cytokines and chemokines in mouse BALF. **(O)** Lung inflammation was evaluated by total cell counts in BALF of mice from different groups (*n* = 5). Representative data shown from two independent experiments. *P*-values are indicated in the panels.

We examined the cytokine and chemokine profiles related to IAV-induced ALI in mouse serum and BALF of different groups. As shown in Figures 5A–C, the levels of MIP-1α and RANTES were significantly lower in the serum of treated mice while the level of G-CSF was increased. In BALF, there were totally six cytokines markedly reduced (IL-1β, IL-6, IL-10, MCP-1, MIP-1α and RANTES) and three cytokines increased (IL-12(p40), IL-12(p70) and G-CSF) after Gal-1 treatment, compared with the untreated group (Figures 5E–L,N). Notably, the levels of IFN-γ in both serum and BALF decreased after Gal-1 treatment although the difference was not significant (Figures 5D,M). In addition, total cells in BALF of IAV/Gal-1 group mice were much less than those in IAV/PBS group, although the number was still much larger than the negative control (Figure 5O). These results indicated that Gal-1 treatment might modulate the expression of inflammatory cytokines and chemokines in IAV-induced ALI.

### Gal-1 Treatment Reduces Viral Replication and Production in the Lungs of Mice Infected With H1N1pdm09

**FIGURE 6:**
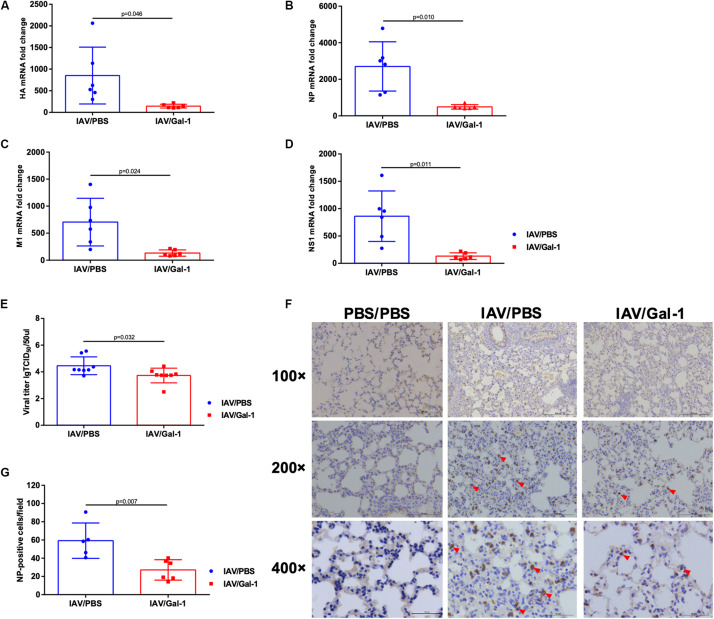
Viral mRNA expression, viral titers and immunohistochemical examination of mice lungs in different groups. **(A–D)** The fold change in viral HA **(A)**, NP **(B)**, M1 **(C),** and NS1 **(D)** mRNA levels in mouse lungs treated with Gal-1 compared with untreated controls (*n* = 6). **(E)** Viral titers in Gal-1-treated mouse lung homogenates compared with untreated controls (*n* = 8). **(F)** Immunohistochemical examination of NP-positive cells in mouse lung sections from different groups at 6 d.p.i. by rabbit polyclonal antibody for influenza A virus NP (original magnification, top panel: ×100, scale bar = 200 μm, middle panel: ×200, scale bar = 100 μm, bottom panel: ×400, scale bar = 50 μm; red arrow head: NP-positive cells). **(G)** NP-positive cells in mouse lungs were quantified by averaging the number of positive stained cells in six randomly selected fields in each section (*n* = 5–6). Representative data shown from at least two independent experiments. *P*-values are indicated in the panels.

As shown in Figures 6A–D, the transcription levels of viral HA, NP, M1 and NS1 mRNA were significantly reduced in Gal-1-treated mice lungs. Similarly, the viral titers were much lower in BALF of Gal-1-treated mice than untreated controls (Figure 6E). To further verify the results, NP expression levels in different groups of mice lungs were assessed. As shown in Figures 6F,G, enumeration of NP positive cells (indicated by red arrow heads) in the lung tissue confirmed significantly less infected cells in mouse lung that received recombinant Gal-1 as a therapeutic compared with IAV/PBS group. Taken together, these results suggested that Gal-1 treatment reduced viral replication and production in the lungs of mice infected with H1N1pdm09.

### Treatment With Recombinant Gal-1 Reduces Weight Loss and Enhances Survival Rate in Mice Against H1N1pdm09 Challenge

**FIGURE 7:**
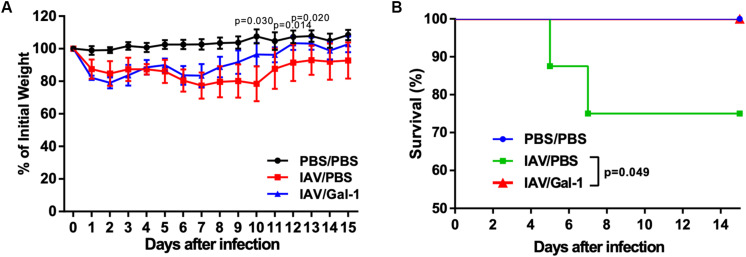
Weight curve and survival curve of mice in different groups. **(A)** Mean changes of body weights from 0 d.p.i. through 15 d.p.i. Body weight was expressed as a percentage of 0 d.p.i. body weight. **(B)** Kaplan–Meier survival curves of different groups of mice (*n* = 13–16). Representative data shown from two independent experiments. *P*-values are indicated in the panels.

Using the infection model described above (Figure 3A), we investigated the effect of recombinant Gal-1 treatment on the final outcomes of H1N1pdm09 infected mice. Mice were weighed and monitored daily for illness and mortality until 15 d.p.i. As shown in Figure 7A, mice receiving Gal-1 treatment were relatively protected against H1N1pdm09 infection, with less weight loss than those in IAV/PBS group. Meanwhile, when comparing changes of body weight in individual mice of each group, we found that more mice in IAV/PBS group continued to get weight loss after 6 d.p.i., when Gal-1-treated mice gradually recovered (Supplementary Figures S1A,B). Furthermore, all Gal-1-treated mice survived while approximately 25% PBS-treated mice died during the observed period, and the difference was significant (Figure 7B). These results demonstrated that Gal-1 treatment offered protection to mice against H1N1pdm09 virus challenge.

### Gal-1 Treatment Reduces H1N1pdm09 Replication and Cell Apoptosis in A549 Cells

**FIGURE 8:**
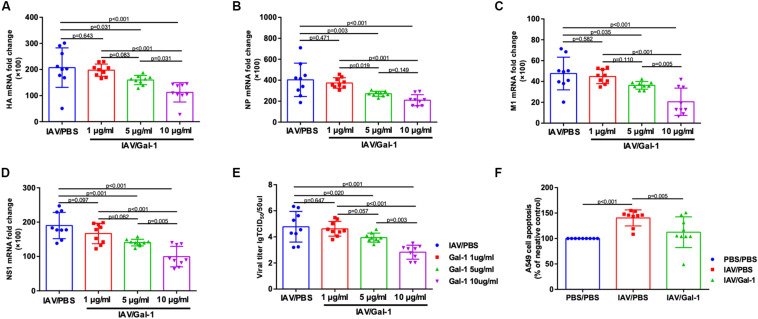
The comparison of viral mRNA expression, viral titers and cell apoptosis in A549 cells between Gal-1 treated and untreated group. **(A–D)** A549 cells were incubated with H1N1pdm09 (1 × 10^3^ TCID_50_) for 2 h and recombinant human Gal-1 (1, 5, or 10 μg/mL) was added. At 72 h.p.i., the fold change in viral HA **(A)**, NP **(B)**, M1 **(C)**, NS1 **(D)** mRNA levels in A549 cells treated with Gal-1 were examined compared with untreated controls. **(E)** Viral titers in Gal-1-treated A549 supernatant compared with untreated controls at 72 h.p.i. (*n* = 9). **(F)** The comparison of apoptotic A549 cell percentage in PBS/PBS, IAV/PBS and IAV/Gal-1 (10 μg/mL) groups by ELISA at 72 h.p.i. (*n* = 9). Representative data shown from three independent experiments. *P*-values are indicated in the panels.

We used cell infection models to explore the effect of Gal-1treatment on viral loads and cell apoptosis in A549 cells. First, the cytotoxic effect of Gal-1 on A549 cells was directly examined through MTT assay and no appreciable cytotoxicity was observed at concentrations ranging from 1 μg/mL to 10 μg/mL (Supplementary Figure S2C). RT-qPCR revealed a significant reduction of viral HA, NP, M1 and NS1 mRNA levels after Gal-1 treatment in a dose-dependent manner, although cells treated with 1 μg/mL Gal-1 have no significant difference with non-treated cells (Figures 8A–D). Similarly, the viral titers were reduced in Gal-1-treated A549 supernatant in a dose-dependent manner and viral titers were the lowest in 10 μg/mL Gal-1 treated group (Figure 8E). Additionally, given that IAV can have typical cytopathic effect (CPE) on A549 cells, which display destroyed structural integrity, shrinkage and rounding, we also relatively quantified cells with CPE in different groups. Relatively reduced CPE in A549 cells was observed in IAV/Gal-1 group at 24 h.p.i., with less percentage of CPE cells in each field, these data were shown in Supplementary Figures S2A,B. For the analysis of cell apoptosis, cell death ELISA were conducted as described above. The level of apoptotic A549 cell percentage was much higher in IAV/PBS group than that in IAV/Gal-1 group treated with 10 μg/mL Gal-1 (Figure 8F). These results indicated that treated with Gal-1 treatment restricted H1N1pdm09 virus replication and ameliorated apoptosis in A549 cells.

## Discussion

Exacerbated outcomes of influenza are often associated with ALI and ARDS, which is characterized by sudden onset of respiratory failure, non-cardiogenic pulmonary edema, and pathologically by bronchiolar wall necrosis, inflammatory infiltration and diffused alveolar injury (Cao et al., 2009). With the realization that these complications are attributed to intrinsic viral tropism and pathogenicity as well as excessive host response (Herold et al., 2015), more and more therapies are attempting to mitigate IAV-induced ALI (Lu et al., 2018). For example, GM-CSF, an important mediator in IAV infection, has been administered to patients with IAV-induced ARDS and is yielding supportive results for a future clinical trial (Herold et al., 2014). Another one, acetylsalicylic acid, an NF-κB inhibitor, is now in a phase II clinical trial for severe influenza, based on its antiviral and additional anti-inflammatory effects (Mazur et al., 2007).

Our current study focused on Gal-1, a ubiquitously expressed animal lectin, which has broad anti-inflammatory effects in various viral and inflammatory diseases (Sundblad et al., 2017; Toudic et al., 2019). The main result of this study was that treating with Gal-1 alleviated acute lung injury, reduced inflammatory infiltration and improved survival rate of H1N1pdm09-infected mice. Meanwhile, our data of *in vitro* experiments showed that Gal-1 treatment decreased IAV mRNA expression and viral titers in a dose-dependent manner.

In our research, inflammatory damage of the lung was existed at all indicated time points including 10 d.p.i, when viral load has been adequately controlled, which strongly indicates that host factors would contribute to lung injury. These results were consistent with previous studies (Zheng et al., 2008; Cui et al., 2016; Alsuwaidi et al., 2017). Additionally, a growing body of clinical and experimental data suggest that the host response to infection is primarily responsible for the pathologic changes observed during respiratory viral infections (Newton et al., 2016; Peteranderl et al., 2016), which can explain our data.

Our findings showed that protein expression level of Gal-1 was increased in mouse serum, BALF and the supernatant of cultured A549 cells after infection. Gal-1 is identified as a proto-type soluble lectin, once synthesized, Gal-1 may remain inside the cell to control intracellular processes, or they may be released to the extracellular space through an unknown pathway (Sundblad et al., 2017). As revealed previously, Gal-1can be released by the infected epithelium (Nita-Lazar et al., 2015b), activated macrophages, and endothelial cells during viral infection or inflammation (Garner et al., 2015), and can serve as an autocrine or paracrine regulator (Lee et al., 2015; Nita-Lazar et al., 2015a). Our results suggested that secreted Gal-1 might have function in H1N1pdm09 infection.

Recently, more and more studies have highlighted the important roles and application of recombinant Gal-1 in virus-induced inflammation and tissue injuries. Aalinkeel et al. (2017) found that Gal-1 treatment reduces human immunodeficiency virus (HIV-1) associated neuroinflammation. Another *in vivo* study showed that treatment with recombinant Gal-1 diminishes HSV-1 induced ocular immunopathological lesions by reducing the production of proinflammatory cytokines and chemokines, indicating an anti-inflammatory and protective effect of Gal-1 on HSV-1 induced injury (Rajasagi et al., 2012). In the current study, we found that pathological changes and wet/dry weight ratio were significantly reduced in mouse lungs after treated with Gal-1. Considering the outcome of inflammatory damage to alveolar epithelial and edema accumulation, the role of Gal-1 treatment might afford an anti-inflammatory and a positive effect on H1N1pdm09-induced ALI. When focusing on the cytokines and chemokines, we found that Gal-1 treatment significantly reduced the production of MIP-1α and RANTES in mouse serum. We also found that IL-1β, IL-6, IL-10, MCP-1, MIP-1α and RANTES in mouse BALF decreased along with reduced BALF cells after Gal-1 treatment. These cytokines are reported to increase in IAV infection and are associated with increased disease severity and affect the outcome of influenza patients (Garcia-Ramirez et al., 2015; Guo et al., 2015; Keshavarz et al., 2019). Among them, MCP-1, IL-1β, IL-6 and MIP-1α are mainly secreted by macrophages during IAV infection (Li et al., 2019). MIP-1 acts as a chemoattractant for monocytes and neutrophils (Jones et al., 2003), as well as, IL-1β and IL-6 increase neutrophil migration to the lung during IAV infection (Camp and Jonsson, 2017). Considering the reduced cells in BAFL, our results suggested Gal-1 treatment might inhibit macrophagocyte and neutrophil recruitment after H1N1pdm09 infection, which was consistent with some previous studies (Fichorova et al., 2016; Law et al., 2020). Additionally, some researches revealed that Gal-1 inhibits recruitment of macrophagocytes by suppressing RANTES (Fichorova et al., 2016), and performs protective effects in ALI by reducing the release of cytokines like IL-6 (Zanon Cde et al., 2015; Newton et al., 2016). Another study found that Gal-1 treatment reduced IL-1β expression induced by nodavirus and performed an anti-inflammatory activity protecting against infection (Poisa-Beiro et al., 2009). These findings were consistent with the reduced RANTES, IL-6 and IL-1β we found and strongly support our results. Currently, there is increasing evidence suggesting that the infiltration of macrophages (Johnston et al., 2012; Huang et al., 2020) and neutrophils (Camp and Jonsson, 2017) are key factors in the pathogenesis of IAV-induced ALI and ARDS, especially at the early stage of disease (Huang et al., 2018), and is accompanied by an increase in the immune-pathological injury of IAV infection (Camp and Jonsson, 2017; Huang et al., 2020). Thus, the reduction of inflammatory cytokines, chemokines and cell numbers after Gal-1 treatment might have a positive effect on H1N1pdm09-induced ALI. Notably, we found decreased levels of IFN-γ in both serum and BALF after Gal-1 treatment although the difference was not significant. As we know, IFN- γ as well as IFN-α/β plays crucial role in antiviral defense (Zhou et al., 2019). On the contrary, they were also implicated in exacerbating inflammation and worsening the outcomes of infectious diseases including IAV infection (Zhou et al., 2019). Similarly, the role of Gal-1 in reducing IFN-γ expression was also reported in EBV and HSV infections and reduced tissue injuries (Gandhi et al., 2007; Rajasagi et al., 2012), these researches also partly support our results.

In the *in vivo* apoptosis analysis of our study, we found that Gal-1 treatment reduced the number of apoptotic cells in mouse lungs. We also found that attenuated chromatin condensation of cells in H1N1pdm09-infected mouse lungs after Gal-1 treatment, providing visualized evidence of reduced cell apoptosis. These results were consistent with the previous reports (Yang et al., 2011; Nita-Lazar et al., 2015a; Perez et al., 2015). Apoptosis is a common phenomenon of IAV-induced ALI (Herold et al., 2015). Accumulated evidence showed that excessive lung inflammation was proved to play a crucial role in the development of apoptosis *in vivo*, which is considered as an underlying mechanism in IAV-induced ALI and ARDS (Chopra et al., 2009). Another study found evidence of infiltrating inflammatory cells in the lung along with an increased number of TUNEL-positive (DNA fragmentation of apoptotic cells) cells in the lung sections with ALI (Chopra et al., 2009), suggesting that reduction of inflammatory infiltration by Gal-1 treatment might ameliorate apoptosis in the lung. To determine the outcome of mice with reduced lung damage by Gal-1 treatment, we monitored the mouse weight and survival rate. As expected, Gal-1 treatment were relatively protected against H1N1pdm09 infection, with less weight loss and more survival were observed in Gal-1 treated mice, indicating reduced ALI by Gal-1 treatment might ameliorate the final outcomes of H1N1pdm09 infected mice.

Besides, reduced viral titers in lungs of mice treated with Gal-1 were found in our study. As mentioned before, host inflammatory response represents a balance between the elimination of virus and immune-mediated tissue injury (Newton et al., 2016). Findings from published studies of IAV-infected animals suggested that an overly exuberant inflammatory response often occurred along with impaired viral clearance, and might result in mortality in IAV infection (Iwasaki and Pillai, 2014). There is some other evidence demonstrating that alleviation of inflammation infiltration the lung is attributed to the reduced levels of viral load during IAV infection (Yang et al., 2011). These reports supported our results, suggesting that alleviated ALI by Gal-1 treatment might relatively maintain IAV clearance. Moreover, some *in vitro* studies found that Gal-1 extracellularly binds to virial envelope or capsid glycoproteins, performing modulatory effects on virus attachment and fusion (Vasta, 2009), such as HIV-1 (Reynolds et al., 2012), DENV-1 (Toledo et al., 2014), and Hendra virus (Levroney et al., 2005). Yang et al. (2011) revealed that Gal-1 could bind to IAV via the interaction between its carbohydrate recognition domains (CRDs) and viral surface N-linked oligosaccharide chains, influencing IAV hemagglutinin binding to receptors. Based on these publishes, we treated A549 cells with Gal-1 *in vitro* and found that Gal-1 treatment reduced viral loads in a dose-dependent manner, although no reduction was found when using the concentration of 1 μg/mL. These results suggested that as a multifunctional protein, the anti-inflammatory role of Gal-1 treatment in alleviating ALI might cooperate with its direct effect on IAV and the precise mechanism remains further study.

We acknowledge some limitations in the present study. We mainly demonstrated the role of Gal-1 in alleviating H1N1pdm09-induced ALI *in vivo* from the perspective of inflammatory regulation, which is considered as the primary feature and cause of IAV-induced ALI (Newton et al., 2016). In our study, we also found reduced viral loads and apoptotic A549 cells treated with Gal-1 *in vitro*, of which the microenvironment was different from that *in vivo* and immune response system was not integrated, prompting that the direct effect on virus and the potential anti-apoptotic activity and of Gal-1 could not be excluded. Further studies are needed to explore the precise mechanism of Gal-1 in regulating cell apoptosis after IAV infection. In addition, although the reduced inflammatory infiltration and changes of related cytokines and chemokines in IAV-induced ALI were found after Gal-1 treatment, further studies to determine the specific immune cells participated in this process are required.

In summary, our findings indicate a potentially effect of Gal-1 treatment on modulating related pro-inflammatory mediators and ameliorating ALI as well as the final outcomes of H1N1pdm09-infected mice. Furthermore, exogenous administration of recombinant Gal-1 may represent a viable adjunctive therapeutic strategy for treating influenza.

## Data Availability Statement

All datasets generated for this study are included in the article/Supplementary Material.

## Ethics Statement

The animal study was reviewed and approved by the Research Ethics Committee of the First Affiliated Hospital, Zhejiang University School of Medicine.

## Author Contributions

JB and YC conceived and designed the experiments. JB, XW, SL, and QZ performed the experiments. JB analyzed the data. JB, QZ, SZ, FY, and YC wrote the original draft, contributed to review and editing.

## Conflict of Interest

The authors declare that the research was conducted in the absence of any commercial or financial relationships that could be construed as a potential conflict of interest.
