# Supplementary material for: Galectin-1 Ameliorates Influenza A H1N1pdm09 Virus-Induced Acute Lung Injury
Source: Front Microbiol. 2020 Jun 12;11:1293. doi: 10.3389/fmicb.2020.01293 (PMC7303544; doi:10.3389/fmicb.2020.01293)
Supplement: Supplementary file 1 [file Data_Sheet_1.PDF]

Supplementary materials:

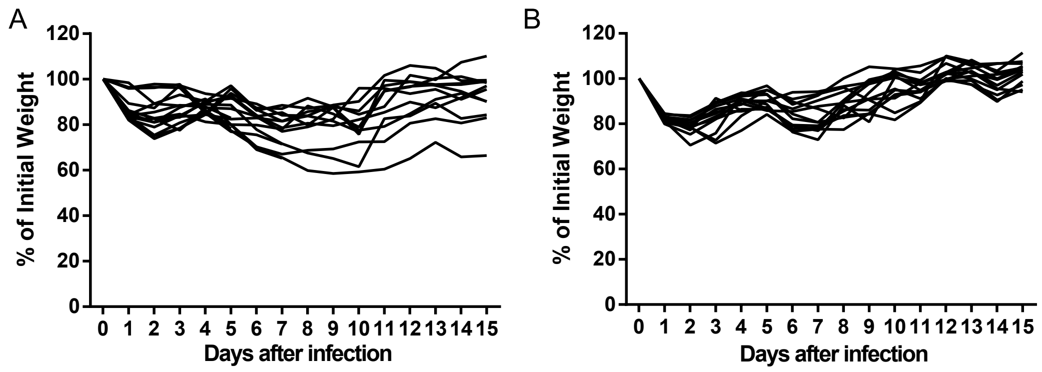

Figure S1. Weight curve of individual mouse in different groups. (A). Changes of body weight in individual mouse from IAV/PBS group. **(B).** Changes of body weight in individual mouse from IAV/Gal-1 group. Body weight was expressed as a percentage of 0 d.p.i. body weight (n=13-16). Representative data shown from two independent experiments.

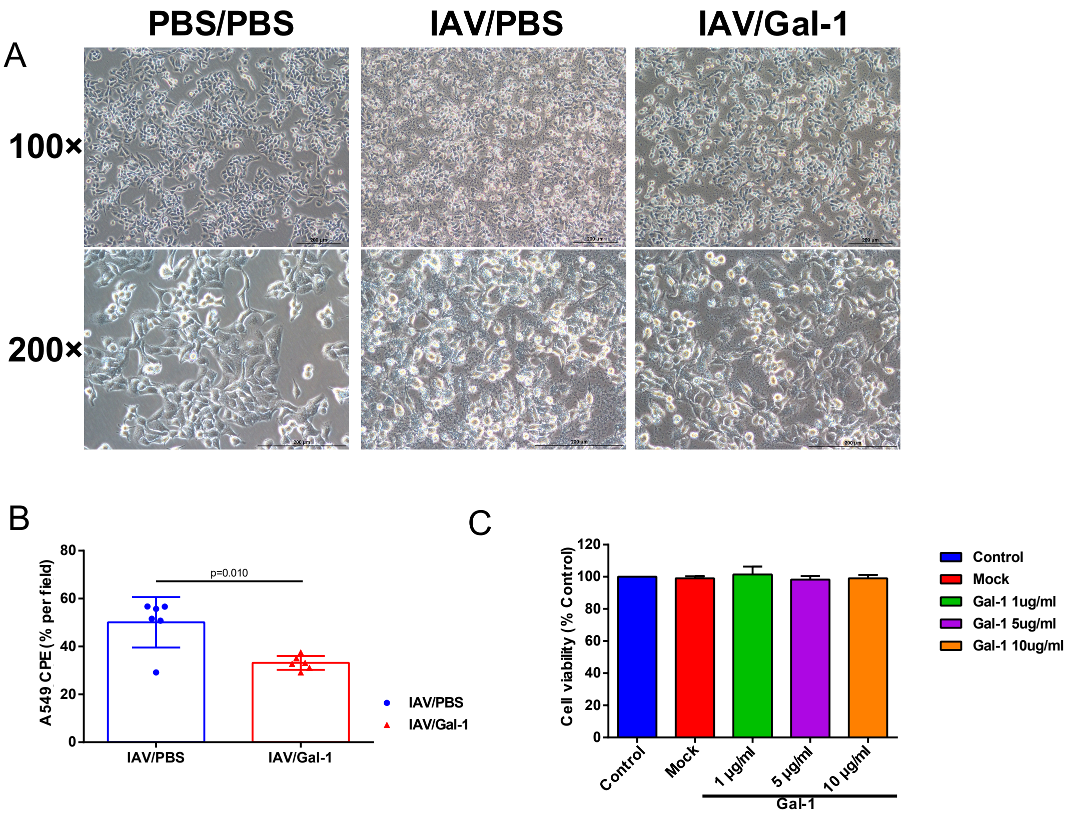

Figure S2. A549 cell pathological change and cytotoxic effect of Gal-1 on A549 cells. (A). H1N1pdm09-induced cytopathic effect of infected A549 cells in different groups. Cells were observed by microscopy and photographed at 24 h.p.i.. (B) Relatively quantification of cells with CPE in different groups at 24 h.p.i.. Data were expressed as an average percentage of CPE cells in each field and six randomly fields were selected. (n=6) (C) MTT assay was performed to examine the cytotoxic effect of Gal-1 (from 1μg /mL to 10 μg/mL) on A549 cells. Data were expressed as (mean OD of treated cells/mean OD of control cells) ×100 (n=4). Representative data shown from at least two independent experiments. P values are indicated in the panels.
